# Cost-effectiveness of adding indoor residual spraying to case management in Afghan refugee settlements in Northwest Pakistan during a prolonged malaria epidemic

**DOI:** 10.1371/journal.pntd.0005935

**Published:** 2017-10-23

**Authors:** Natasha Howard, Lorna Guinness, Mark Rowland, Naeem Durrani, Kristian S. Hansen

**Affiliations:** 1 London School of Hygiene and Tropical Medicine, London, United Kingdom; 2 HealthNet-TPO, Peshawar, Pakistan; 3 Faculty of Health Sciences, University of Copenhagen, Copenhagen, Denmark; Institute of Collective Health, Federal University of Bahia, BRAZIL

## Abstract

**Introduction:**

Financing of malaria control for displaced populations is limited in scope and duration, making cost-effectiveness analyses relevant but difficult. This study analyses cost-effectiveness of adding prevention through targeted indoor residual spraying (IRS) to case management in Afghan refugee settlements in Pakistan during a prolonged malaria epidemic.

**Methods/Findings:**

An intervention study design was selected, taking a societal perspective. Provider and household costs of vector control and case management were collected from provider records and community survey. Health outcomes (e.g. cases and DALYs averted) were derived and incremental cost-effectiveness ratios (ICERs) for cases prevented and DALYs averted calculated. Population, treatment cost, women’s time, days of productivity lost, case fatality rate, cases prevented, and DALY assumptions were tested in sensitivity analysis. Malaria incidence peaked at 44/1,000 population in year 2, declining to 14/1,000 in year 5. In total, 370,000 malaria cases, 80% vivax, were diagnosed and treated and an estimated 67,988 vivax cases and 18,578 falciparum and mixed cases prevented. Mean annual programme cost per capita was US$0.56. The additional cost of including IRS over five years per case prevented was US$39; US$50 for vivax (US$43 in years 1–3, US$80 in years 4–5) and US$182 for falciparum (US$139 in years 1–3 and US$680 in years 4–5). Per DALY averted this was US$266 (US$220 in years 1–3 and US$486 in years 4–5) and thus ‘highly cost-effective’ or cost-effective using WHO and comparison thresholds.

**Conclusions:**

Adding IRS was cost-effective in this moderate endemicity, low mortality setting. It was more cost-effective when transmission was highest, becoming less so as transmission reduced. Because vivax was three times more common than falciparum and the case fatality rate was low, cost-effectiveness estimations for cases prevented appear reliable and more definitive for vivax malaria.

## Author summary

We conducted a cost-effectiveness analysis of adding malaria prevention to routine malaria diagnosis and treatment in Afghan refugee settlements in Pakistan during a five-year malaria epidemic. We found that malaria incidence peaked at 44 per 1,000 in year 2 and declined to 14 per 1,000 in year 5, with an average annual programme cost per capita of US$0.56 in 2015 currency. Cost per case prevented averaged US$88 (US$111 for vivax, US$442 for falciparum), per death prevented averaged US$316,734, and per DALY averted averaged US$601. The additional cost of including IRS over five years per case prevented was US$39 (US$50 for vivax and US$182 for falciparum malaria case prevented) and per DALY averted was US$266. While our cost-effectiveness results were relatively high, when compared with internationally recognised cost-effectiveness thresholds both prevention and case management were highly cost-effective, indicating the relevance of an integrated approach for epidemic malaria control and global malaria elimination.

## Introduction

Despite almost two decades of radically increased public funding and significant gains, malaria control remains challenging as resistance develops to existing insecticides and antimalarials [1–4]. The World Health Organization (WHO) recommends an integrated control approach of early diagnosis and treatment, vector control, epidemic surveillance and response, and improved information systems [1, 5, 6]. Despite these advances, financing of malaria control for populations displaced by crises and conflicts is by definition limited in scope and duration [7, 8]. Cost-effectiveness analyses, by comparing costs and consequences of alternative interventions, can increase effective programme management, but are particularly difficult to conduct in complex, resource-constrained settings. Thus, no studies were found exploring full operational costs or cost-effectiveness of integrated malaria control in refugee settings or including epidemic transmission, and minimal published cost-effectiveness research was found for malaria prevention in South Asia [9, 10].

Parts of Afghanistan and former North-West Frontier Province (NWFP), now Khyber-Pakhtunkhwa Province, Pakistan remain malaria endemic [11–14]. Breakdown of control infrastructure during decades of conflict led to a high annual malaria burden for Afghanistan during 1990–2010 [8, 13, 15]. Inflows of over 3 million Afghan refugees to Pakistan in the 1980s and 1990s led to increased transmission in newly-settled areas [16–19]. Refugee populations were particularly vulnerable, due to being predominantly non-immune and settled in areas prone to Anopheline breeding [17, 19–22]. To inform future refugee programme design and implementation, data from the 1990–1995 epidemic were retrospectively evaluated, as primary healthcare services in settlements changed relatively little over this chronic emergency period.

This study aimed to determine whether adding targeted malaria prevention using indoor residual spraying (IRS) to case management using quality-assured microscopy and national first-line treatment was a better use of limited resources than case management alone during five years of epidemic malaria control in Afghan refugee settlements in Pakistan. Objectives were to: (i) calculate costs of the integrated control programme and vector control and case management components; (ii) determine costs per malaria case and death prevented, year of healthy life gained (YHLG), and disability-adjusted life-year (DALY) averted; and (iii) calculate incremental cost-effectiveness ratios (ICERs) for integrated control relative to case management alone.

## Methods

### Study design

Following Consolidated Health Economic Evaluation Reporting Standards (CHEERS) guidelines [23], an intervention study design was selected to answer the research question ‘Is adding a vector control intervention to existing malaria case management for refugees cost-effective?’ taking a societal perspective. Programme implementation costs, outcomes (i.e. cases and deaths prevented, YHLG, DALYs), and cost-effectiveness (ICERs) were calculated.

### Study site and population

The study population resided in Afghan refugee settlements near the Afghanistan-Pakistan border. In year 0, a population of almost 2.5 million Afghan refugees was supported by 193 Basic Health Units (BHUs) in over 100 settlements in NWFP (Table 1). By year 5, this had dropped to 1.2 million refugees supported by 71 BHUs. As the refugee population declined and NGOs reduced activities, numbers of operational BHUs reduced by nearly two-thirds.

**Table 1:** Epidemiology, programme effectiveness, and cost-effectiveness by year.

**Epidemiological indicators**	**Year 0**	**Year 1**	**Year 2**	**Year 3**	**Year 4**	**Year 5**
Population	2,402,726	2,386,726	1,832,077	1,541,577	1,298,006	1,236,325
*P*. *vivax* cases	65,410	85,560	63,909	54,096	28,898	15,815
*P*. *falciparum* cases	16,121	12,984	16,495	7,376	3,941	1,895
Mixed falciparum cases	829	282	253	133	15	122
*Total cases*	*82*,*360*	*98*,*826*	*80*,*657*	*61*,*605*	*32*,*854*	*17*,*832*
**Vector control response (%)**						
Number of settlements sprayed with malathion	0	64 (100)	76 (100)	39 (51)	19 (56)	13 (45)
Number of settlements sprayed with lambda-cyhalothrin	0	0 (0)	0 (0)	33 (43)	15 (44)	13 (45)
Number of settlements sprayed with permethrin	0	0 (0)	0 (0)	5 (6)	0 (0)	3 (10)
*Total settlements sprayed*	*0*	*64*	*76*	*77*	*34*	*29*
**Case management response**						
total BHUs	193	190	180	155	101	71
per 10,000 population	0.80	0.80	0.98	1.01	0.78	0.57
**Effectiveness indicators**	**Year 0**	**Year 1**	**Year 2**	**Year 3**	**Year 4**	**Year 5**
**Cases prevented**						
*P*. *vivax*	..	13,979	19,406	21,181	11,769	1,653
*P*. *falciparum*/mixed	..	3,106	6,144	7,754	1,087	487
Total cases		17,085	25,550	28,935	12,856	2,140
**Deaths prevented**	..	22	44	55	8	3
**Years healthy life gained**						
*P*. *vivax*	..	460	638	696	387	54
Discounted *P*. *vivax*	..	460	619	656	354	48
*P*. *falciparum*	..	949	1,878	2,370	332	149
Discounted *P*. *falciparum*	..	949	1,823	2,234	304	132
**DALYs averted**	..	2,511	3,756	4,253	1,890	315
**Cost-effectiveness indicators**	**Year 0**	**Year 1**	**Year 2**	**Year 3**	**Year 4**	**Year 5**
***Cost per case prevented***						
*P*. *vivax*	..	US$142	US$88	US$85	US$106	US$539
*P*. *falciparum*/mixed	..	US$641	US$278	US$233	US$1,152	US$1,829
Total cases	..	US$116	US$67	US$63	US$97	US$416
***Cost per death prevented***	..	US$90,224	US$39,182	US$32,886	US$257,576	US$116,415
**Cost per YHLG**	..	US$1,412	US$679	US$590	US$1,741	US$4,383
**Cost per discounted YHLG**		US$1,412	US$700	US$626	US$1,903	US$4,948
**Cost per DALY averted**	..	US$792	US$455	US$426	US$662	US$1,033

Refugee populations were non-immune and constantly changing, having originated in areas where malaria was previously controlled and engaging in considerable cross-border movement. Refugee settlements were sited on marginal land and housing was rapidly constructed from mud-brick, lacking piped water or sewerage. The United Nations High Commissioner for Refugees (UNHCR) provided integrated housing, education, and health services for refugees. The UNHCR malaria control programme responded to a late 1980s malaria epidemic and operated for over fifteen years. Implementers were governmental Project Department for Health (PDH) and local and international non-governmental organizations (NGOs). HealthNet International (now HNTPO), a specialist technical NGO, conducted research and provided technical support, quality-assured malaria microscopy training, monthly field laboratory monitoring, malaria surveillance, and vector control targeting and evaluation.

### Intervention

The UNHCR-subsidised intervention consisted of: (i) case management through strengthening malaria diagnosis and treatment at BHUs in all settlements during years 0–5; and (ii) vector control using annual indoor residual spraying (IRS) in a sub-set of higher-incidence settlements during years 1–5. Case management, conducted by BHU health-workers, consisted of diagnosis by quality-assured microscopy and treatment of malaria cases according to national guidelines [19]. Microscopy was the only diagnostic method used. Continuous training and quality checks, through bimonthly monitoring of each BHU laboratory (i.e. experienced microscopists checked a randomly-selected series of negative and positive malaria slides from each microscopist), maintained a diagnostic accuracy of above 98% [19]. Rapid diagnostic tests were not available at test sites, but it was deemed unlikely by investigators that their usage would have improved the level of accuracy achieved. Positive cases received a 3-day course of chloroquine (CQ) as first-line treatment, with primaquine (PQ) administered as a gametocytocidal drug for falciparum malaria and as a 5-day course for vivax malaria, though this was later abandoned as a trial demonstrated insufficiency for radical treatment [24]. UNHCR procured chloroquine locally and primaquine internationally. All cases were recorded for surveillance purposes and asked to return for a follow-up slide, with most doing so, and treatment failures receiving sulphadoxine-pyrimethamine (SP). National guidelines have since changed for confirmed falciparum malaria to more effective and more expensive SP-artesunate therapy [25], and this substitution was modelled in sensitivity analysis.

IRS vector control was conducted by refugee workers, supervised by implementing partners, in an annual campaign held before the onset of peak annual transmission (July-August). Refugee settlements were spatially discrete, ranging from approximately 5,000 to 30,000 population, and separated from local Pakistani villages. Settlements were generally densely populated, and though density sometimes varied, it was straightforward for malaria control staff to identify spatially the perimeters of each settlement and houses within them, all of which were eligible for IRS. IRS targeting was based on a threshold reported malaria incidence rate per settlement of above 5 falciparum cases per 1,000 person-years or 30 vivax cases per 1,000 person-years in the previous year. Pumps and insecticide were donated by UNHCR. The organophosphate insecticide malathion and the pyrethroid lambdacyhalothin were used for IRS (Table 1). All services were provided free to end-users.

### Effectiveness calculations

Effectiveness measures used were cases prevented, deaths prevented, YHLG, and DALYs averted. YHLG were reported, both because DALYs were very low due to low recorded mortality and for comparison with studies not reporting DALYs. Malaria incidence per 1,000 population was estimated from BHU-diagnosed malaria cases, as 87% of refugees surveyed reported using BHUs for healthcare [17, 26]. Population figures were taken from biannual UNHCR records and crosschecked with HNTPO data, NGO family registrations, and spraying records, but potentially over-represented due to population mobility. As population estimates affected incidence calculations, a reduced population set was included in sensitivity analysis.

#### Cases prevented by vector control

Cases prevented by vector control were calculated in sprayed settlements as ‘the number of actual cases’ minus ‘the number of cases that would have occurred in the absence of spraying’. Unsprayed settlements were used for controls and matched with sprayed settlements with similar populations and incidence rates from the same district, using randomised controlled trial principles. As some unsprayed settlements had lower incidence rates, which could have underestimated cases prevented, a higher transmission reduction was tested in sensitivity analysis.

#### Cases prevented by case management

Cases prevented by case management could not be calculated readily, as no counterfactual settlements without case management existed. Thus, the worst-case scenario was used in which case management prevented no additional cases and thus had no impact on transmission, with higher estimates modelled in sensitivity analysis.

#### Deaths prevented

Deaths prevented were calculated as the product of the number of falciparum cases prevented multiplied by the case fatality rate (CFR). This enabled comparison across vector control and case management and derivation of YHLG. Vivax CFR was estimated as zero. A falciparum CFR of 0.71% (i.e. 44 deaths out of 6,210 falciparum cases) for Afghan refugees was obtained from two years of mortality data in study settlements in Hangu district [27]. As household deaths were seldom reported, not directly attributable to malaria, and CFR data were collected during epidemic conditions, both a higher CFR and lower non-epidemic CFR were tested in sensitivity analysis.

#### Years of healthy life gained (YHLG)

Years of healthy life gained (YHLG) summed morbidity and mortality gains from cases prevented. Mean days of illness per episode, obtained by household survey, were 12 for vivax and 18.4 for falciparum. Mean age at death for falciparum, obtained from BHU records, was 16.4 years. Age-disaggregated life expectancy data were obtained from WHO life tables for Afghanistan over the study time-period [28] and used for calculating years of life lost. Morbidity gains per case averted were calculated as mean days ill multiplied by percentage of nonfatal cases (i.e. 1 minus CFR). Mortality gains per case averted were calculated as: ‘life expectancy at age of death’ minus ‘mean age at death’ multiplied by 1 minus CFR. A 3% discount rate was used, to capture present valuation of future benefit or harm [29], as its common usage in other studies improved comparability [30–32]. YHLG were obtained by dividing day results by 365.

#### DALYS averted

DALYS averted were calculated for falciparum and vivax combined according to WHO methods, with 3% discounting, 0.053 disability weighting for moderate infectious disease, and uniform age weighting [33, 34], as: ‘years of life lost to malaria mortality plus years of malaria-related disability in the absence of the intervention’ minus ‘years of life lost to malaria mortality plus years of malaria-related disability in the presence of the intervention’. Major DALY assumptions (i.e. discount rate, age weighting, disability weighting, life expectancy, CFR) were tested in the sensitivity analysis.

### Cost calculations

Using a standard ingredients approach, total costs were calculated over five years for the whole control programme and for vector control and case management components individually. *Provider cost data* were collected from UNHCR, HNTPO and four implementing partners providing healthcare services covering 80% of settlements. Shared provider costs, associated with both vector control and case management (i.e. malaria inspectors and supervisors, administrative and finance staff, general health-staff, overheads, storage, transport) were allocated per programme component. *Household cost data* were estimated by facility exit survey. The survey was conducted by two male CHWs, experienced in interviewing, of 623 malaria outpatients at four BHUs in Naguman, Kotki, and Azakhel settlements, purposively selected to represent lower, middle, and higher-income households, to calculate weighted average household costs. Data collected on: (i) malaria species diagnosed; (ii) travel and waiting times for BHU providers; (iii) travel and waiting times for non-BHU providers; (iv) payments at non-BHU providers; and (v) time and productivity lost from illness were used to estimate direct and indirect household costs per malaria episode.

#### Total annual case management costs

Total annual case management costs were estimated as: (‘provider cost per case’ plus ‘household cost per case’) multiplied by ‘number of cases diagnosed and treated annually’. Case management costs per strain and per year were calculated as: (‘number of positive slides recorded’ multiplied by ‘cost per case diagnosed and treated’) plus (‘number of negative slides recorded’ multiplied by ‘cost per slide’), with mixed cases assumed to incur falciparum costs and negative results costed as: slide plus reagent plus ‘microscopist time per slide’. Provider costs for case management summed direct costs from all participating providers. Specific costs were laboratory technicians, microscopes, slides and reagents, antimalarials, training, and monitoring. Shared costs were allocated as described above for vector control. Household costs for case management summed direct and indirect costs for service-users, which were estimated per episode by the exit survey then multiplied by numbers of cases to estimate total annual costs. Direct costs were costs incurred through treatment. Indirect costs were costs incurred through travel and the value of time lost from regular activities, due to illness, and due to caring for those ill. Service-user costs per case were: ‘direct costs’ plus ‘indirect costs’ plus ‘carer costs’ plus ‘productivity lost to morbidity or mortality’.

Direct household costs were: ‘average per-case test and treatment costs for non-BHU providers’ plus ‘average per-case travel costs’. Estimated travel costs for patients and carers were: ‘travel costs to and from a facility’ multiplied by ‘number of journeys per person per episode’. As polypharmacy was common among private providers, meaning malaria patients received non-essential tests and drugs they would not have incurred without malaria infection, these were included as direct costs.

Indirect household costs were: (‘travel time to and from facilities’ multiplied by ‘number of journeys per person per episode’ plus ‘time per person spent in facilities waiting and consulting’ plus ‘additional non-productive time spent ill’) multiplied by ‘time value for patients and carers’. Time spent ill was estimated from survey data as 12 days for vivax and 18.4 days for falciparum malaria. As this was potentially overestimated, a lower estimate was tested in sensitivity analysis. Time was valued as ‘time in days’ multiplied by ‘daily wage’ multiplied by ‘% in paid employment’ using the average daily wage of US$1.65 paid by PDH and estimating that 34.7% of adult refugees (i.e. 65% of men, 1% of women) earned wages based on HNTPO interview data [35]. As this estimation undervalued women’s unpaid domestic work, higher estimations were tested in sensitivity analysis. Carers were normally women or unemployed householders, thus replacement-cost valuation using the generalist daily wage paid to refugee labourers (i.e. US$1.65) was selected. While non-working children could not be disaggregated, malaria was most frequent in working ages. A cost per malaria death could have been calculated, using an adapted human capital approach to estimate potential life-long productivity losses, but was not for ethical reasons as data did not allow for willingness-to-pay estimations.

#### Total annual vector control costs

Total annual vector control costs summed provider and service-user costs associated with vector control. Costs for participating providers were entomologists, spray-personnel, spray pumps, protective gear, and insecticide. Implementing partners provided specific and shared cost data (e.g. overheads, vehicles, personnel) from expenditure records, budget files and staff interviews. As malaria control was part of integrated health-service delivery, shared support cost allocation differed by provider. Cost allocation approaches were: (i) personnel costs multiplied daily wages by estimated time in post; (ii) annual training was calculated as (number of trainees for NWFP programme/number of trainees for all programmes) multiplied by (total training costs); (iii) transport and overheads were split by number of operational sectors weighted by numbers of malaria staff or similar indicator of programme size; and (iv) monitoring costs were estimated from HNTPO interviews. Household costs associated with vector control, estimated by provider interview, summed time lost to women’s unpaid work during house preparation, time waiting for IRS/drying, and post-spraying house reorganisation, based on replacement cost of a domestic worker. Household costs were very small and thus treated as zero for simplicity.

#### Annual per-capita case management costs

Annual per-capita case management costs were calculated by dividing total case management costs by total recorded settlement population.

#### Annual per-capita vector control costs

Annual per-capita vector control costs were calculated by dividing total vector control costs by the total recorded population of settlements sprayed.

All costs were converted to US$2015 constant prices using Pakistan’s national GDP deflator and International Monetary Fund statistics [36–38]. Capital costs were annualised using the World Bank discount rate for Pakistan of 10% and expected useful lifespan (i.e. BHU buildings and microscopes at 20 years, vehicles at 10 years, and computers, photocopiers, and spray pumps at 5 years).

### Cost-effectiveness calculations

#### Cost per case prevented by vector control

Cost per case prevented by vector control was calculated as: ‘total programme and household costs of vector control and case management’ divided by ‘total number of malaria cases prevented’. No cases were prevented by case management in the main analysis, so costs were calculated as zero. The incremental cost-effectiveness ratio (ICER) for cases prevented by vector control was then calculated as difference in cost divided by difference in effect: (‘total programme and household costs of vector control and case management’ minus ‘total programme and household costs of case management alone’) divided by (‘number of cases prevented by the whole programme’ minus ‘number of cases prevented by case management alone’). Costs and ICERs for malaria deaths prevented, per YHLG, and per DALY averted were calculated similarly. The WHO cost-effectiveness threshold for DALYs of 3 times Pakistan’s GDP per capita in year 0, i.e. US$1,436 (range US$537-US$3,864), was used because preference elicitation data were unavailable and it remains an established threshold [39, 40]. However, given criticisms of the WHO threshold by Shillcutt and others [41, 42], Woods *et al*’s threshold values for Pakistan, i.e. US$87-US$669, were also compared [43].

### Sensitivity analysis

A univariate sensitivity analysis was conducted, as probabilistic analysis would have been difficult without cost and effect distributions. Inputs for major assumptions of population, malaria treatment costs, valuation of women’s time, days of productivity lost to illness, CFR, cases prevented, and DALY assumptions were varied and resulting costs compared with effectiveness outcomes. To test the effects on the ICER for cases prevented of:

1. increasing falciparum treatment costs, a higher estimate using SP+Artesunate was used;
2. reducing population size, an additional annual incidence set was calculated using a population of one-half recorded size;
3. varying cases prevented by vector control, an increased rate of 50% was tested [17, 44];
4. increasing cases prevented by case management above zero, rates of 30% and 50% were tested [45];
5. reducing days of productivity lost to malaria illness, lower estimates from Nepal (i.e. 7.9 days for vivax, 10.9 days for falciparum) were used [46];
6. reducing valuation of women’s time, two additional daily rates of $1.00 and $0.00 were used.

To test the effects of varying DALY assumptions on the ICER for DALYs averted:

- the 3% discount rate was compared with a rate of 5% and 10% [47];
- uniform age weighting was compared with non-uniform weighting;
- disability weighting of 0.053 for an acute moderate infection episode was compared with 0.005 for a mild episode and 0.254 for post-acute consequences as estimated for the 2010 Global Burden of Disease [34];
- historic Afghan life expectancy was compared with a 2014 Afghan life table [28]; and
- the 0.71% CFR was compared with a possible non-epidemic estimate of 0.05% (an estimated midrange CFR for Pakistan for 2003–2013 [48]) and a doubled epidemic estimate to account for potential underreporting of deaths, as there was no required death registration.

### Ethics statement

Local ethics approval for operational research was provided by the UNHCR Pakistan office. Approval for retrospective data analysis was provided by the research ethics committee of the London School of Hygiene and Tropical Medicine (reference 5508). All patient data were anonymised prior to inclusion.

## Results

### Epidemiology and response

**Fig 1:**
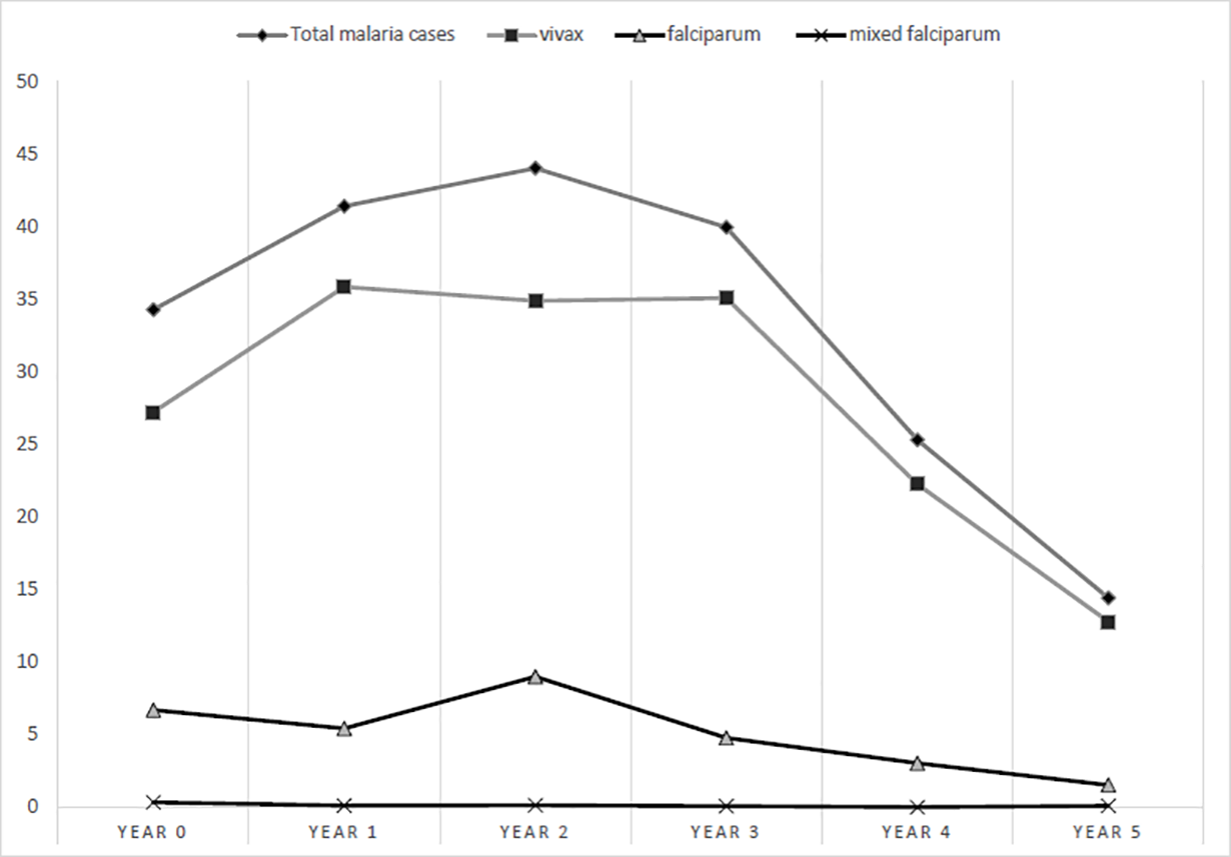
Malaria incidence per thousand in refugee settlements, Year 0 to Year 5.

Fig 1 shows that malaria incidence in refugee settlements rose from 34 per 1,000 person-years in year 0 to a peak of 44 per 1,000 person-years in year 2, then declined steadily to 14 per 1,000 person-years in year 5. Table 1 shows annual population, malaria incidence, and programme responses. At baseline in year 0, 193 health facilities provided case management for 2.4 million refugees (0.8/10,000 population). The number of facilities peaked in years 2–3 with 1.0/10,000, decreasing to 0.6/10,000 in year 5. From year 1 onwards, high-incidence settlements also received IRS, peaking in year 3 with 50% (77/155) of BHU catchment settlements covered and reducing to 41% (29/71) of remaining settlements in year 5. Programme effectiveness and cost-effectiveness results are reported for years 1–5 only, as year 0 did not include IRS.

### Effectiveness analysis

#### Cases and deaths prevented

Table 1 shows that over years 1–5, an estimated 67,988 vivax cases (annual average 13,598), 18,578 falciparum and mixed-infection cases (annual average 3,716), and 132 deaths (annual average 26) were prevented through IRS.

#### YHLG

Days of healthy life gained per case prevented were 12 for *P*. *vivax*, 111 for *P*. *falciparum*. Per case estimates were multiplied by cases prevented and then by 365 to obtain a total 2,235 YHLG for vivax prevention (annual average 447) and total 5,678 YHLG for falciparum prevention (annual average 1,136). Discounted YHLG totalled 2,138 (annual average 428) for vivax prevention and 5,443 (annual average 1,089) for falciparum prevention (Table 1).

#### DALYs averted

Disability-adjusted life-years averted were estimated as 0.147 per case prevented for total cases, accounting for vivax and falciparum morbidity and mortality. This was then multiplied by cases prevented to obtain a total of 12,725 DALYs averted (annual average 2,545) during years 1–5 (Table 1).

### Cost analysis

#### Total costs

**Fig 2:**
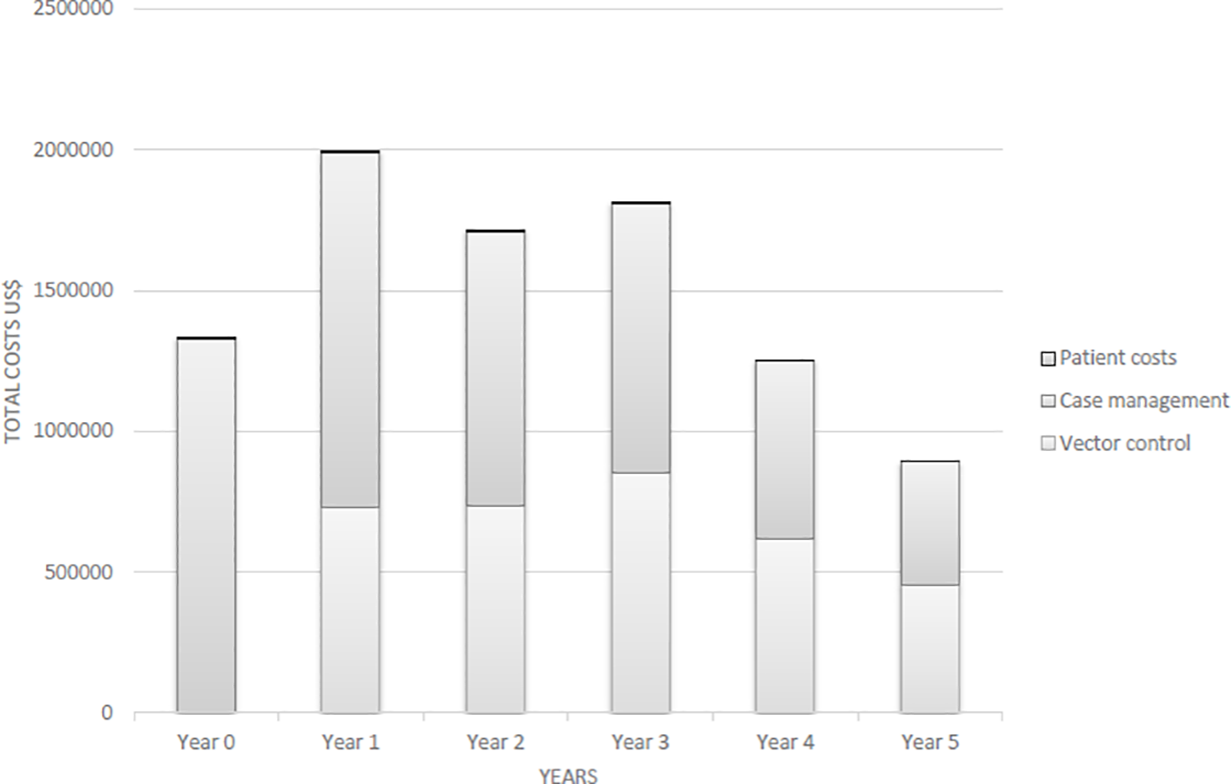
Total programme costs by year.

Programme costs totalled US$8.9 million (US$1.5 million annual average) for the full six years (years 0–5) and US$7.7 million (also US$1.5 million average) for the five years included in cost-effectiveness analysis. UNHCR funded approximately 56% overall with remaining costs divided between HNTPO (i.e. 12% overall) and NGO service-providers (i.e. 32% overall). HNTPO and NGO service-providers’ proportional contributions shifted, from 6% and 50% respectively in year 0 to 26% each in year 5. Fig 2 shows an overall decline in costs from US$1.3 million at baseline to US$890,623 in year 5, with two peaks in years 1 and 3. Case management was proportionately higher at US$5.6 million (62%), decreasing from US$1.3 million annually (100%) at baseline to US$438,765 annually (49%) in year 5. Vector control totalled US$3.4 million (38%), peaking in year 3 at US$852,477 (47%). Service-user case management expenditures totalled US$27,861 (0%), decreasing with the epidemic curve from US$7,025 (1%) at baseline to US$1,117 (0%) in year 5.

#### Component costs

The costliest vector control components were personnel and insecticide at US$2.9 million (85%) total. An increase in vector control costs in year 3 was associated with increased insecticide costs due to initiation of lambda-cyhalothrin for IRS in half of settlements sprayed. The costliest case management component was personnel at US$3.7 million (67%). Locally purchased antimalarials and imported primaquine, at US$272,283 (5%) and US$35,692 (1%) respectively, were minor components. Provider transport increased as a share of costs comparatively over time, from US$153,078 (12%) at baseline to US$83,374 (19%) in year 5. Service-users spent 22% more treating falciparum than vivax malaria (i.e. US$16,899 versus US$10,962 respectively), though these were minor components (0%) of overall societal costs.

#### Per capita costs

The mean annual cost per capita for the full programme was US$0.56, ranging US$0.36–0.76 over five years. Mean annual costs per capita for vector control and case management separately were US$0.23 and US$0.33 respectively. For vector control, this ranged from US$0.0–0.34, highest in year 3. For case management, this ranged from US$0.23–0.40, highest in year 0 (US$0.36) and year 3 (US$0.40).

#### Per-case costs

Provider costs per case diagnosed and treated averaged US$14.98 for vivax and US$14.93 for falciparum, with the largest component cost being US$10 for staff. Household costs per case were estimated from survey data as US$10.83 for vivax and US$18.91 for falciparum. Given low mortality rates, the largest household component cost per case was productive time lost to illness at US$8.22 for vivax and US$12.63 for falciparum.

### Cost-effectiveness analysis

Table 2 compares cost-effectiveness of the integrated programme versus case management alone for a population of 100,000 and includes costs and ICERs of events averted averaged over the full five-year analysis period (years 1–5), and years 1–3 and 4–5 separately, to compare changes over time. In a model population of 100,000, the *cost per case prevented* averaged US$88 (US$77 in years 1–3 and US$143 in years 4–5). For vivax, cases prevented averaged US$111 (US$103 in years 1–3 and US$160 in years 4–5). For falciparum, cases prevented averaged US$442 (US$331 in years 1–3 and US$1,361 in years 4–5). *Cost per death* prevented averaged US$316,734 (US$45,635 in years 1–3 and US$191,715 in years 4–5). *Cost per YHLG* averaged US$1,011 (US$806 in years 1–3 and US$2,323 in years 4–5). *Cost per DALY averted* averaged US$601 (US$524 in years 1–3 and US$972 in years 4–5).

**Table 2:** Effects, costs, and incremental cost-effectiveness ratios of adding vector control to case management in refugee settlements in Pakistan over five years, and disaggregating years 1–3 and 4–5, in US$2015.

**Effects per 100,000 population**	**Integrated programme**	**Case management**	**Comparison**
	*n = 100*,*000*	*n = 100*,*000*	*n = 100*,*000*
Vivax cases prevented	835	..	835
Falciparum/mixed cases prevented	209	..	209
All cases prevented	1,044	..	1,044
Deaths prevented	0.29	..	0.29
YHLG	91	..	91
DALYs averted	153	..	153
			
**Costs per 100,000 population**	**Average cost-effectiveness ratio**		**Comparison**
**Costs of events averted over 5 years**	**US$**	**US$**	**ICER US$**
Programme costs per 100,000	92,250	51,406	
Cost per vivax case prevented	111	..	50
Cost per falciparum/mixed case prevented	442	..	182
Cost per case prevented	88	..	39
Cost per death prevented	316,733	..	140,234
Cost per YHLG	1,011	..	448
Cost per DALYs averted	^a,^*601	..	^a^^,^**266
**Costs of events averted in years 1–3**	**US$**	**US$**	**ICER US$**
Programme costs per 100,000	95,643	55,420	
Cost per vivax case prevented	103	..	43
Cost per falciparum/mixed case prevented	331	..	139
Cost per case prevented	77	..	32
Cost per death prevented	45,635	..	19,192
Cost per YHLG	806	..	339
Cost per DALYs averted	^a,^*524	..	^a^^,^**220
**Costs of events averted in years 4–5**	**US$**	**US$**	**ICER US$**
Programme costs per 100,000	84,539	42,284	
Cost per vivax case prevented	160	..	80
Cost per falciparum/mixed case prevented	1,361	..	680
Cost per case prevented	143	..	71
Cost per death prevented	191,715	..	95,825
Cost per YHLG	2,323	..	1,161
Cost per DALY averted	*972	..	^a^^,^*486

*Below WHO cost-effectiveness threshold of US$1,435.86 (3*US$478.62 for Y0 Pakistan GDP per capita) per DALY averted.

**Highly cost-effective at <US$478.62 (1*Y0 Pakistan GDP per capita) per DALY averted. GDP = gross domestic product; <GDP per capita is ‘very cost-effective’, 1–3*GDP per capita is ‘cost-effective’, >3*GDP per capita is ‘not cost-effective.

‘ ^a^ Below Woods *et al*’s cost-effectiveness threshold for Pakistan of US$87–669.

The additional cost of including IRS in a model population of 100,000 averaged over five years per case prevented was US$39 (US$33 in years 1–3 and US$69 in years 4–5). For vivax cases prevented this was US$50 (US$43 in years 1–3 and US$80 in years 4–5). For falciparum cases prevented this was US$182 (US$139 in years 1–3 and US$680 in years 4–5). The additional cost averaged over five years per death prevented was US$140,234 (US$19,192 in years 1–3 and US$95,825 in years 4–5). The additional cost averaged over five years per YHLG was US$448 (US$339 in years 1–3 and US$1,161 in years 4–5). The additional cost averaged over five years per DALY averted was US$266 (US$220 in years 1–3 and US$486 in years 4–5). Adding IRS to routine case management was thus ‘highly cost-effective’ using the WHO threshold of US$479 per DALY averted (i.e. Y0 Pakistan GDP per capita) when averaged over five years and in years 1–3. This reduced in years 4–5, but remained ‘cost-effective’ at the WHO threshold of US$1,436 per DALY averted (i.e. 3 times Y0 Pakistan GDP per capita). The additional costs of adding IRS to case management per DALY averted were cost-effective over all time-periods using Woods *et al*’s threshold of US$87–669.

### Sensitivity analysis

Table 3 shows results of varying treatment costs, population, cases prevented, days of productivity lost, time valuation, and DALY assumptions. Increasing falciparum treatment costs to account for ACT, reducing days of productivity lost, and reducing valuation of women’s time had no notable effect on the ICER. Reducing the population by one-half increased the ICER from US$39 to US$78. Increasing cases prevented by vector control by 50% reduced the ICER to US$26, while increasing cases prevented by case management increased the ICER to US$56 for a 30% increase and US$78 for a 50% increase. For DALY assumptions, increasing the discount rate increased the ICER for DALYs averted from US$266 to US$399. Changing to non-uniform age weighting increased the ICER to US$477. Varying disability weighting had little effect, while varying life expectancy had no effect on the ICER. Lowering the CFR to a non-epidemic average increased the ICER to US$3,914. Doubling the CFR lowered the ICER to US$133.

**Table 3:** Sensitivity to selected parameters of the societal incremental cost-effectiveness ratio (ICER) in US$2015 of cases prevented or DALYs averted.

Parameter: Cases prevented^&^	ICER	Parameter: DALYs averted^&^	ICER
*Study ICER for cases prevented*	*39*	*Study ICER for DALYs averted*	*266*
***Cost of Pf treatment*** *(US$0*.*83)*			
Increased by 150%	39	***DALY assumptions*:**	
Increased by 300%	39	**Discount rate (3%)**	
***Population size (2*,*402*,*726)***		5%	399
Reduced by 50%	78	10%	611
***Cases prevented by vector control (63%)***		**Age weighting (uniform K = 0)**	
Increased by 50%	26	Non-uniform	477
***Cases prevented by case management (0%)***		**Disability weighting (0.053)**	
30% of total cases prevented	56	0.005	323
50% of total cases prevented	78	0.254	320
***Time valuation for women (US$1*.*65/day)***		**Life expectancy (2000)**	
US$1.00/day	39	2014 table	266
US$0.00/day	39	**Case fatality rate (0.71%)**	
***Days productivity lost to illness (12*.*0; 18*.*4)***		Lower non-epidemic (0.05%)	3,914
Pv 7.9 days	39	Higher epidemic (1.42%)	133
Pf 10.9 days	39		

^&^Actual parameter values used for the study are in parentheses.

## Discussion

### Primary findings

This study is the first to model the cost-effectiveness of adding vector control to case management during an epidemic, in a co-endemic vivax-predominant setting. It is one of the first to estimate costs per case averted and first to estimate costs per DALY averted by IRS in South Asia, where IRS has traditionally dominated as a means of malaria prevention [10, 49].

Annual indicators showed increasing then decreasing programme efficiency over six years as population and malaria incidence increased then declined. Similarly, the rise and sustained fall in household costs reflected fewer service-users due to population decline, reduced numbers of BHUs, and falling incidence. IRS prevented approximately 266% more vivax than falciparum cases (i.e. 67,988 vivax versus 18,578 falciparum), due to the dominance of *P*. *vivax* in the area. Conversely, IRS enabled approximately 154% more YHLG from falciparum than vivax prevention (i.e. 5,678 falciparum YHLG versus 2,235 vivax YHLG), because falciparum prevention was worth more YHLG (i.e. 0.30 years for falciparum versus 0.03 for vivax). DALYs averted, which included both vivax and falciparum, were relatively low due to the low estimated falciparum CFR.

IRS appeared to improve horizontal equity (providing equal access to all those with equal needs) because it cost less than case management for households. Household case management costs, though insignificant within overall programme terms, were likely difficult for low-income service-users. Direct household costs were low because IRS was provided free to service-users and most attended BHUs, thus receiving free treatment to which they were able to walk. However, indirect case management costs, such as income lost due to illness, were a concern for low-income households. Additionally, those who attended private providers could pay significant amounts for less reliable diagnosis and treatment.

Adding IRS appeared highly cost-effective in epidemic conditions and less so as cases and CFR reduced. This intervention supported refugees during prolonged epidemic conditions in refugee settlements, but cost-effectiveness can be compared with other programmes using costs to the health system (per capita), per case diagnosed and treated, and per case or DALY averted [50]. For example, health system costs per capita for five years (calculated by dividing total programme costs by total population in the study area), were US$0.73 for the full programme, US$0.62 for case management, and US$0.84 for vector control. These compared favourably to health system costs for malaria control found by Shretta *et al* globally (US$2013 2.50), for sub-Saharan Africa (US$2013 1.21–3.47), and for South Asia (Afghanistan US$2013 1.34; India US$2013 0.30–9.39; Nepal US$2013 0.45–1.36) [50]. Average cost per patient diagnosed and treated (US$14.95) was similar to findings by Hansen *et al* for microscopy diagnosis and treatment in moderate (US$2013 10.64) and low (US$2013 22.38) transmission settings in Afghanistan and by Bualombai *et al* (US$2013 13.23) in Thailand, though higher than by Davis *et al* (US$2013 4.36) in Papua New Guinea per child vivax case diagnosed and treated [51].

Due to low morbidity and mortality compared with high-endemicity predominantly falciparum malaria settings, costs per event averted were relatively high, making cost-effectiveness results for IRS higher than in many endemic settings [49, 52, 53]. For example, average cost per case averted (US$88) was higher than findings by Smithuis *et al* (US$2013 16.54) or Kamolratanakul et al (US$2009 2.7) for IRS with DDT in Myanmar and the Thai-Myanmar border respectively, but lower than findings by Bhatia *et al* (US$2013 126.39) for IRS with deltamethrin in Gujarat [49, 51]. Given DDT was considerably cheaper than other IRS insecticides, this is perhaps unsurprising. Average cost per DALY averted (US$601) was considerably higher than findings by Yukich *et al* (US$2008 119–132) for IRS in Mozambique and KwaZulu-Natal [52]. The programme was more effectively compared with South Asian programmes, as malaria transmission patterns were similar. However, while Bhatia *et al* reported costs per case averted, no other South Asian studies were found reporting costs of cases or DALYs averted through IRS [9, 51]. It is possible that researchers in South Asia have tended to avoid calculating DALYs for malaria prevention interventions as relatively low incidence and proportionally high vivax transmission means fewer cases, lower mortality, and thus elevated costs per DALY averted. However, study results indicate that cost-effective results per DALY averted are still feasible in the region. Additionally, while it would have been useful to compare cost-effectiveness with interventions in epidemic situations, such evidence remains minimal as already noted by Worrell *et al* in 2004 [54].

As incidence continued to fall in the refugee settlements, the malaria control strategy evolved away from targeted IRS to insecticide-treated net (ITN) social marketing and then targeted free distribution of ITNs to communities at highest malaria risk [15, 55]. ITNs have generally been found to be more cost-effective than IRS, with a review by White *et al* finding the median ICER per DALY averted from a provider perspective was US$2009 27 for ITNs (range US$2009 8–110 from 15 African studies) versus US$2009 143 for IRS (range US$2009 135–150 from two southern African studies) [49]. It is possible that the incremental cost of adding prevention using ITNs would be lower than for IRS but this cannot be assumed given the effectiveness of IRS for transmission control in South Asia and should be compared in the same setting [26]. The policy of chloroquine treatment for *P*. *vivax* infection has not changed in Pakistan and vivax remains the predominant malaria species [56].

### Implications

Despite malaria incidence being relatively lower than in sub-Saharan Africa, and vivax predominating, ICER results showed that adding malaria prevention with targeted IRS to routine case management was cost-effective using both WHO’s aspirational and potentially overly-generous threshold of three times GDP and Woods *et al*’s more conservative range for Pakistan of US$87–669 [40, 43]. Results can potentially provide lessons outside South Asia as malaria control progresses toward elimination. Considerations additional to cost will become increasingly important as countries transition to elimination and costs per event averted likely increase [3, 4, 57]. Additionally, in mixed *Plasmodium* species settings, *P*. *falciparum* is more easily reduced by vector control, leaving *P*. *vivax* as the majority species [17]. Therefore, cost-effectiveness analyses of malaria control interventions in low-endemicity, unstable, and epidemic settings could become increasingly important.

Methodologically, accurate cost-effectiveness analysis for *P*. *falciparum* depends primarily on the accuracy of CFR calculations. The CFR estimate used was taken from a camp survey and BHS data [27]. Because no formal system for death notification existed for refugees, who had no incentive to report deaths, reported causes of death were not confirmed and CFR estimates were subject to unverifiable assumptions. Estimates for vivax and falciparum cases prevented were more reliable because they were based on quality-assured microscopy of actual cases and on differences in clinical incidence between matched sprayed and unsprayed camps during the study period [16, 17]. Vivax malaria was three times more common than falciparum malaria and CFR was zero for vivax. Thus, cost-effectiveness analysis for vivax made fewer assumptions than for falciparum and is likely to be more accurate. This study can therefore be considered a more definitive cost-effectiveness analysis for vivax than for falciparum malaria.

Programmatically, changes to organisational structure could increase sustainability. Cost distributions for both case management and vector control indicated personnel as the major cost component. As HNTPO provided technical support in maintaining low malaria endemicity levels, its average costs increased as incidence fell. Thus, staff rationalisation appeared feasible over the long term by strengthening managerial skills and widening the responsibilities of local personnel and broadening the skill base of technical personnel (e.g. training malaria specific diagnosticians and health-workers to take a wider role in public health programmes, such as diagnosis and treatment of other diseases). Excepting insecticide and antimalarials, remaining cost components were minimal and their reduction would have had minimal impact on total costs.

### Limitations

Calculations were subject to several dataset limitations and assumptions. First, calculation of effectiveness indicators was subject to methodological assumptions. The use of matched unsprayed settlements was the best control group available retrospectively and the limited regional data did not allow for epidemic or other modelling to improve estimates further. Data testing indicated this comparison was valid. Second, use of private providers by refugees could underestimate case numbers while vivax recrudescence could cause overestimation of numbers yet underestimation of intervention effectiveness. Third, low numbers of falciparum cases could have reduced accuracy of resulting service-user costs. Fourth, recall bias could have affected the accuracy of some cost data, though the proportions of estimated costs that relied on participant recall (e.g. household costs) were relatively minimal overall. Fifth, Woods *et al*’s cost-effectiveness threshold calculations used QALYs rather than DALYs and compared results directly with WHO thresholds, which seems justified for decision-making given the large margins of error likely in both approaches. Woods *et al* themselves call for further research on realistic cost-effectiveness thresholds for low and middle-income countries [43]. Finally, cost per death prevented was most sensitive to CFR, and respective indicators should be interpreted cautiously.

## Conclusions

While the cost-effectiveness of IRS varied depending on indicators used, the fact remains that many cases and deaths were prevented and the prolonged epidemic was controlled over the study period. Though case management remains a key component of malaria control, this study shows how prevention–in this case using IRS–can be an important and cost-effective component of malaria transmission control even in a moderate endemicity, low mortality setting.
